# Single-Molecule SERS Discrimination of Proline from Hydroxyproline Assisted by a Deep Learning Model

**DOI:** 10.1021/acs.nanolett.5c01177

**Published:** 2025-04-17

**Authors:** Yingqi Zhao, Kuo Zhan, Pei-Lin Xin, Zuyan Chen, Shuai Li, Francesco De Angelis, Jian-An Huang

**Affiliations:** † Research Unit of Health Sciences and Technology, Faculty of Medicine, University of Oulu, Aapistie 5 A, 90220 Oulu, Finland; ‡ Research Unit of Disease Networks, Faculty of Biochemistry and Molecular Medicine, University of Oulu, Aapistie 5 A, 90220 Oulu, Finland; § Biocenter Oulu, University of Oulu, Aapistie 5 A, 90220 Oulu, Finland; ∥ The Biomimetics and Intelligent Systems (BISG) research unit, Faculty of Information Technology and Electronic Engineering, University of Oulu, 90220 Oulu, Finland; ⊥ Istituto Italiano di Tecnologia, Via Morego 30, 16163, Genoa, Italy

**Keywords:** Post-translational modification, SERS, CNN, Plasmonic nanopore, Hydroxyproline and proline, Single molecule

## Abstract

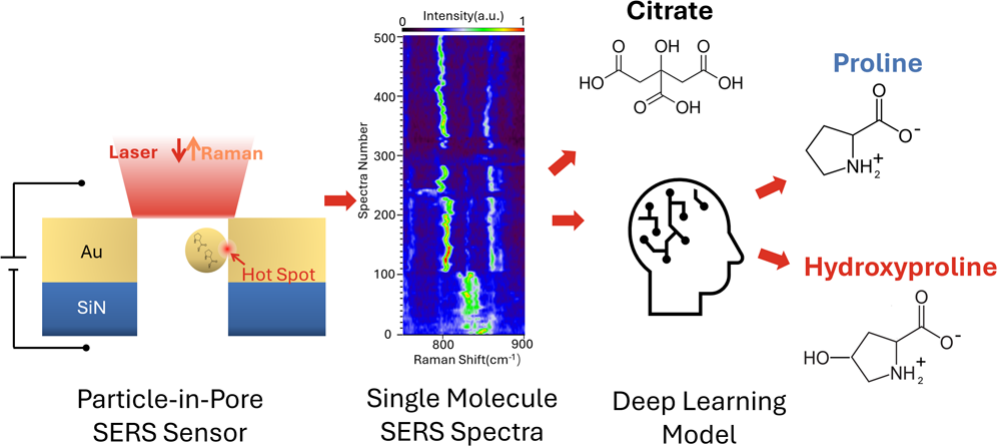

Discriminating low-abundance hydroxylation is a crucial and unmet need for early disease diagnostics and therapeutic development due to the small hydroxyl group with 17.01 Da. While single-molecule surface-enhanced Raman spectroscopy (SERS) sensors can detect hydroxylation, subsequent data analysis suffers from signal fluctuations and strong interference from citrates. Here, we used our plasmonic particle-in-pore sensor, occurrence frequency histogram of the single-molecule SERS spectra, and a one-dimensional convolutional neural network (1D-CNN) model to achieve single-molecule discrimination of hydroxylation. The histogram extracted spectral features of the whole data set to overcome the signal fluctuations and helped the citrate-replaced particle-in-pore sensor to generate clean signals of the hydroxylation for model training. As a result, the discrimination of single-molecule SERS signals of proline and hydroxyproline was successful by the 1D-CNN model with 96.6% accuracy for the first time. The histogram further validated that the features extracted by the 1D-CNN model corresponded to hydroxylation-induced spectral changes.

Post-translational modification (PTM) plays a fundamental role in regulating cellular processes by significantly affecting the protein structure and dynamics. In particular, prolyl 4-hydroxylation catalyzes the addition of a hydroxyl group to proline residues in collagens, which regulates hypoxia-inducible transcription factor (HIF) and activates hundreds of genes in an oxygen-dependent manner to drive cancers.^1,2^ This important discovery was awarded the Nobel Prize in Physiology or Medicine in 2019. As a critical regulator of cancer development, HIF hydroxylation suppresses HIF-1α and constrains tumor growth. However, if impaired, for example, by the loss of the von Hippel-Lindau protein, which is common in clear cell renal carcinoma, HIF accumulates and unleashes its oncogenic potential.^3,4^ HIF hydroxylation can serve as a potential biomarker of tumor behavior assessment and therapy response,^5^ as well as therapeutic targeting.^6^ Yet, the current detection methods based on mass spectrometry and antibodies are facing challenges in detecting low-abundance and site-specific PTM.^7,8^ Therefore, single-molecule analysis of trace amounts of PTM biomarkers in human biofluids is of great significance for early-stage disease diagnosis, low-abundance protein studies, and therapeutic development.^9^

Mass spectrometry is currently the dominant technology for hydroxylation analysis. However, its sensitivity is constrained by spontaneous nonspecific oxidations and sample loss, which usually requires 10^6^–10^8^ molecular copies, and therefore it is challenging to detect low-abundance hydroxylation. Nanopore resistive pulse sensing has emerged as a promising single-molecule detection method, which distinguishes biomolecules based on current changes as they pass through a nanopore. Using this technique, single-molecule PTM detection has been demonstrated for phosphorylation,^10^ acetylation,^11^ propionylation,^12^ glycosylation,^13^ nitration, and oxidation.^14^ However, the difference in molecular structure (−OH group with 17.01 Da) between hydroxylated proline and proline is too small to induce sufficient current changes in nanopores for reliable detection, making resistive pulse analysis of single-molecule hydroxylation particularly challenging.

Surface-enhanced Raman spectroscopy (SERS) is another promising tool for label-free PTM detection due to its high sensitivity and ability to provide molecular structural information.^15^ Similar to the nanopore resistive pulse sensors, significant research efforts have focused on using plasmonic colloids to detect PTMs with large Raman cross sections and distinct SERS spectral features, for example, phosphorylation,^16,17^ nitration,^18^ and oxidation.^19^ Yet, few SERS-based detections have been reported for small PTMs with small Raman cross sections, including hydroxylation, due to the difficulties in obtaining sufficient SERS spectra for analysis or the limitation of SERS system sensitivity.^20,21^ Another problem caused by the small PTM Raman cross section is that the peptide backbone signal can screen or merge the PTM spectra.^1^ As a result, there has been no report on the SERS detection of hydroxylation at the single-molecule level.

In contrast to the above SERS methods, our recent plasmonic particle-in-pore sensor demonstrated SERS spectroscopic detection of the 20 amino acids and peptides at the single-molecule level.^22,23^ The sensor generated a single gap-mode plasmonic hot spot with single-molecule sensitivity between a gold nanoparticle and the nanopore side wall, as shown in Figure 1(a). When a molecule enters the hot spot, part of it is located in the small hot spot and excited,^24^ thereby generating SERS signals from the corresponding molecule moiety and avoiding the backbone signal coverage. However, challenges in the single-molecule SERS detection of the hydroxylation lie in the spectra interpretation due to the fluctuation and blinking of the single-molecule spectra. What is worse, the citrate surfactants on the nanoparticle surface or solution may interfere with the SERS data analysis of single-molecule hydroxylation.

**Figure 1:**
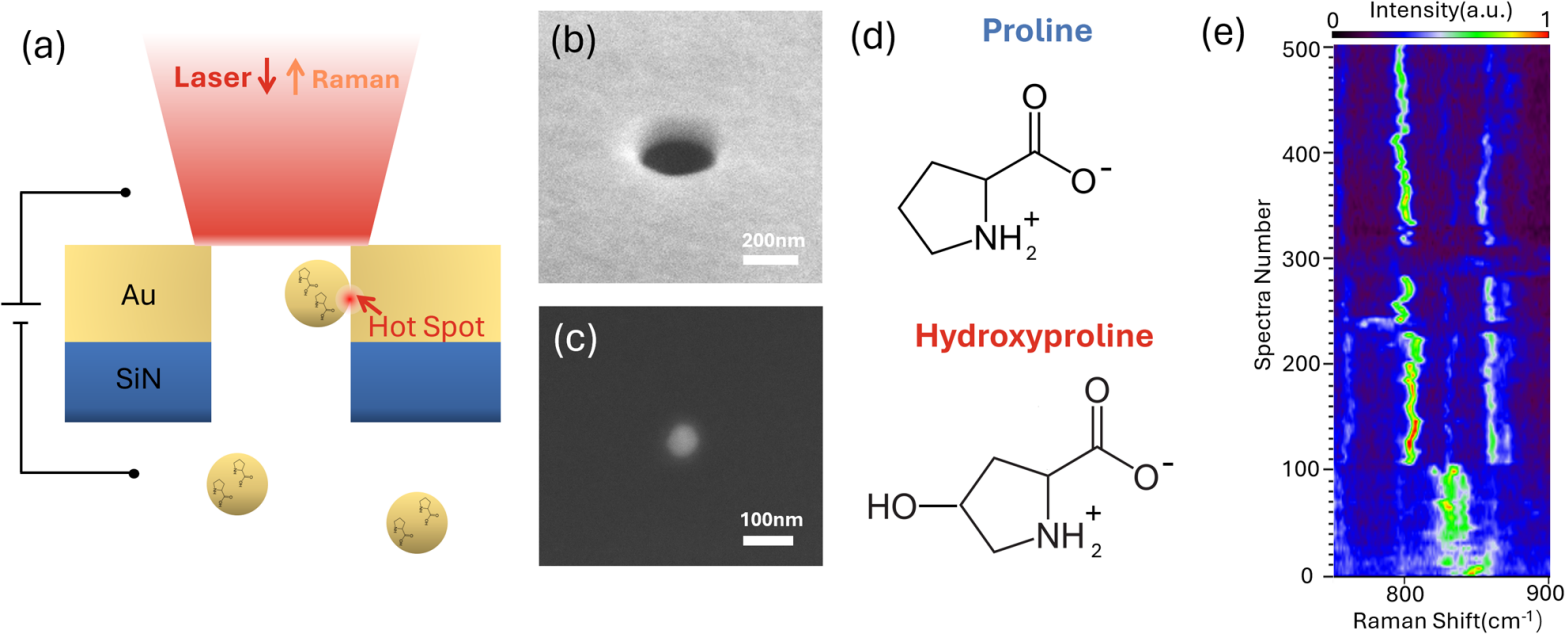
(a) Schematic illustration of the particle-in-pore sensor that allows single AuNPs loaded with analytes to be trapped in a gold nanopore to generate a plasmonic hot spot upon laser illumination. (b) SEM image of the nanopore. (c) SEM image of the AuNP. (d) The molecular structures of Pro and Hyp at pH 5.5 in solution. (e) Typical fluctuating single-molecule SERS spectra of Pro. The color bar is the normalized signal intensity in arbitrary units.

In this study, we report the single-molecule SERS discrimination of proline (Pro) and hydroxyproline (Hyp) using the particle-in-pore sensor, the occurrence frequency histogram of SERS peaks, and a deep learning model of a one-dimensional convolutional neural network (1D-CNN). To analyze the highly dynamic single-molecule data, we employed occurrence frequency histograms to filter out intensity fluctuations caused by nanoparticle morphology variation while preserving structural information for molecular discrimination. Using these histograms, we investigated the substitution of citrates on gold nanoparticles (AuNPs) with analytes to minimize citrate interference in spectral interpretation and benefit the training of the 1D-CNN model. As a result, the 1D-CNN model achieved more than 96% accuracy in both testing and post evaluation. Finally, we used 1D gradient-weighted positive feature visualization to validate discrimination based on Raman bands corresponding to hydroxylation-induced spectral changes in the pyrrolidine ring. To the best of our knowledge, this is the first successful discrimination of an amino acid with and without hydroxylation, one of the most challenging PTMs for SERS, at the single-molecule level, laying the solid foundation for the detection of low-abundance HIF hydroxylation.

## Sensor Fabrication and Measurement

Figure 1(a) presents a schematic illustration of the particle-in-pore sensor, which consists of a gold nanopore array with a 200 nm diameter that was drilled on a 100 nm gold film supported on a free-standing SiN membrane with a silicon frame by a focused ion beam. The detailed fabrication process is recorded in Supplementary Note 1. Figure 1(b) shows an SEM image of a typical nanopore. The SiN membrane chip is encapsulated into a microfluidic device with top and bottom chambers, across which an electric potential is applied. The analyte, Pro or Hyp, is physically adsorbed onto a 50 nm diameter AuNP (Figure 1(c)) before being injected into the bottom reservoir. The negatively charged AuNPs are then driven through the nanopore by the cross-membrane electric potential. A 785 nm laser illuminates the nanopore from above, generating an optical force that pushes the nanoparticle toward the nanopore sidewall to generate the gap-mode hot spot formed between the AuNP and the nanopore sidewall with single-molecule sensitivity.^22^ During the particle trapping period, the analyte could diffuse into the hot spot, where it is excited to produce single-molecule SERS spectra.

A monolayer of Pro or Hyp was attached to AuNPs by incubation in a 5% PBS buffer containing Pro and Hyp at pH 5.5. The concentration of molecules required to achieve monolayers capping the AuNP surfaces was determined by empirical values of maximum solvent accessibilities of amino acids^25^ and nanoparticle surface area; the details are discussed in the Supplementary Note 2. At pH 5.5, both molecules exist in their zwitterionic form, as shown in Figure 1(d). The purchased AuNPs were initially stabilized by a citrate layer, which acted as a surfactant. Upon incubation in a Pro or Hyp solution, the citrate was replaced by amino acids due to their higher binding affinity to AuNPs,^26,27^ as discussed in Supplementary Note 3. Typical single-molecule SERS spectra of Pro show fluctuating SERS peaks, as plotted in Figure 1(e).

## Molecular Profiling from Single-Molecule SERS Spectra

**Figure 2:**
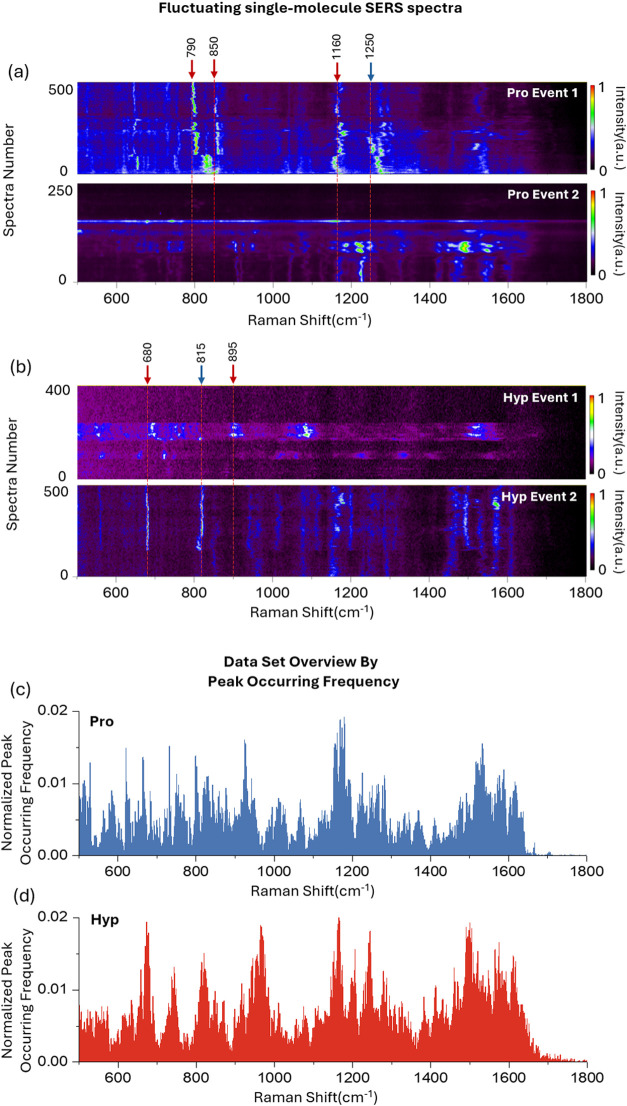
Waterfall plot of typical single-molecule SERS spectra of (a) Pro and (b) Hyp trapping events shows the vibrating feature of single-molecule SERS spectra. The color bars indicate the normalized signal intensity in arbitrary units. The red arrow indicates that the band shows in only one trapping event, while the blue arrow indicates that the band shows in both trapping events. Distribution histograms of normalized peak occurrence frequency of (c) Pro and (d) Hyp on AuNP with 48 h monolayer incubation. The number of spectral events contained in the histograms for Pro and Hyp is 11,002 and 9769, respectively.

The explanation of single-molecule spectra is challenging due to fluctuations and blinking features.^28^Figure 2(a) and (b) present typical SERS waterfall plots generated by the sensor, showing two distinct trapping events for each molecule. In a single trapping event, only a subset of vibrational modes is typically excited. For instance, in Pro event 1, three bands near 790 cm^–1^ (δ_COO^–^_),^21^ 850 cm^–1^ (ρ_CH_2__),^29^ and 1160 cm^–1^ (t_CH2_)^29^ are observed but absent in event 2, while the band near 1250 cm^–1^ (ω_CH2_)^29^ appears in both. Similarly, for Hyp, the band at 815 cm^–1^ (t_CH2_)^21^ appears in event 1, but the 680 cm^–1^ (δ,ω_COO^–^_)^21^ is present in event 2, while a band at 895 cm^–1^ (cit)^30^ is shown in a few spectra in event 1, but exhibits long trapping in event 2. This variation arises because when a AuNP is trapped near the nanopore sidewall, the resulting hot spot is only a few angstroms in size—comparable to the molecular dimensions. As a result, only specific portions of the molecule reside within the hot spot, leading to selective excitation of vibrational bands based on molecular position and orientation.^31^ Given that molecules diffuse on the nanoparticle surface and the nanoparticle still has partial Brownian motion while trapped in the nanopore, the particle-in-pore sensor captures different molecular moieties in each trapping event or spectrum. By collecting large data sets, the system effectively reconstructs a more complete molecular profile.

Single-molecule SERS bands exhibit fluctuations in both the position and intensity. The peak intensity varies due to factors such as the distance between the nanoparticle and the nanopore sidewall, hot spot size, molecular position, and orientation, making it less reliable for distinguishing molecular structural differences. However, despite peak position fluctuations, the central position can be statistically extracted from a sufficiently large data set, providing insights into structural changes. By analyzing peak positions and their frequency of occurrence across multiple trapping events, the general spectral characteristics can be determined.^32^

The peak frequency histogram is an efficient tool to obtain a general understanding of the large amount of single-molecule SERS data.^32^ We quantified the peak occurring frequency at each Raman shift and plotted normalized histograms for Pro and Hyp, as shown in Figure 2(c) and (d). Raw data were preprocessed through cosmic-ray-removal, normalization, and baseline correction. The number of peak occurrences was then divided by the total number of effective spectra (details provided in the Supplementary Note 1). The overall spectral profiles of Pro and Hyp are similar due to their structural resemblance, although they differ in specific peak positions. By displaying peak occurrence frequency and position (Raman shift), the histograms highlight single-molecule SERS bands at high spectral resolution that would otherwise overlap in multimolecule SERS spectra or powder Raman spectra. For instance, in the Pro histogram in Figure 2(c), four CH_2_ rocking bands appear near 825 cm^–1^, 844 cm^–1^, 855 cm^–1^, and 878 cm^–1^,^29^ whereas these bands may merge into two or three in powder Raman spectra (as shown in Figure S4 in the Supporting Information) or multimolecule SERS spectra.^20,21^ Although not all vibrational modes are excited in a single trapping event, the particle-in-pore sensor captures detailed spectral information that might be lost in conventional multimolecule SERS spectra. Due to the lack of reference for single-molecule proline/hydroxyproline SERS spectra, precise peak assignment in a conventional way is not straightforward, besides the interference of citrate on AuNP, making it even difficult to discriminate analytes only based on the histogram. However, the histogram of peak occurrence frequency was also proven to be a useful tool for analyzing citrate substitution and determining the valid data set for 1D-CNN model training, which will be discussed below.

## Citrate Substitution

The presence of surfactants on AuNPs complicates precise peak assignment in single-molecule SERS data obtained from the particle-in-pore system. Due to the system’s extreme sensitivity, even residual citrate—used as a stabilizing surfactant for AuNPs—can no longer be ignored. To investigate citrate substitution, we analyzed AuNPs incubated in the analyte solution (Pro and Hyp in this work) for 0 and 48 h and then measured the peak occurrence frequency. Based on our previous protocol, a 48 h incubation time was chosen, as it provides a balance between sufficient substitution, colloidal stability, and efficiency.^23^ The analyte concentration was adjusted to ensure monolayer coverage of the AuNPs. Additionally, citrate detection by the particle-in-pore sensor using AuNPs with 1/8 monolayer Hyp coverage and a 24 h incubation time was conducted as an example of insufficient citrate substitution.

**Figure 3:**
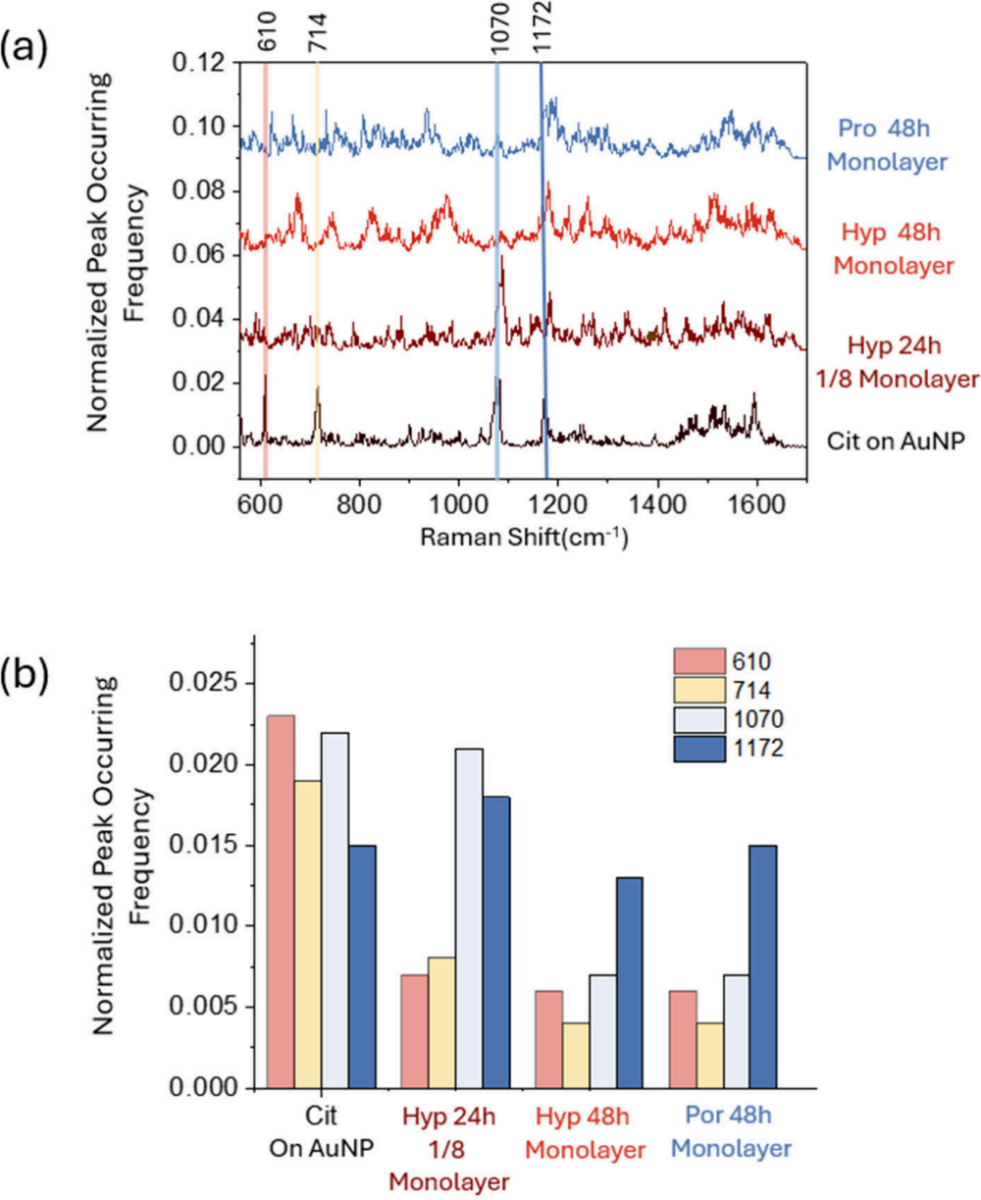
(a) The normalized peak occurring frequency generated in particle-in-pore sensors using AuNPs with citrate capping (black curve), 24 h incubation in solution with Hyp concentration for 1/8 monolayer (dark red curve), 48 h incubation in solution with Hyp concentration for a monolayer (red curve), and 48 h incubation in solution with Pro concentration for a monolayer (blue curve). Four characteristic peaks of citrate-capped AuNPs at 610 cm^–1^, 714 cm^–1^, 1070 cm^–1^, and 1172 cm^–1^ are indicated by red, yellow, light blue, and blue guiding lines. (b) Histograms of the band height at 610 cm^–1^, 714 cm^–1^, 1070 cm^–1^, and 1172 cm^–1^, generated by AuNPs with citrate capping, 24 h incubation in solution with Hyp concentration for 1/8 monolayer, 48 h incubation in solution with Hyp concentration for a monolayer, and 48 h incubation in solution with Pro concentration for a monolayer.

In the absence of analytes, AuNPs remained capped with citrate with the corresponding peak occurrence frequencies shown in Figure 3(a) (black curve). Four high-intensity bands at 610 cm^–1^ (δ_COO_),^33^ 714 cm^–1^ (δ_OCO_),^30^ 1070 cm^–1^ (ν_CO_),^33^ and 1172 cm^–1^ (δ_COO_)^33^ were chosen to indicate citrate presence. The observed peak occurrence frequencies qualitatively correlate with the molecular population adsorbed on the AuNP surface, as shown in Figure 3(b). Under sufficient substitution conditions by the 48 h monolayer incubation, the 1070 cm^–1^ peak remains visible but its intensity significantly decreases. In the case of insufficient substitution, the 610 cm^–1^ and 714 cm^–1^ bands are reduced compared to those at the 0 h incubation case but remain higher than those in the 48 h incubation condition. Due to overlap with the Pro/Hyp vibrational modes, the 1172 cm^–1^ band does not exhibit a clear trend. After 48 h, the citrate bands become almost negligible, indicating minimal interference. However, this does not confirm complete citrate displacement, as analyte adsorption onto AuNPs occurs through a dynamic process. Even when the analyte concentration is sufficient to form a monolayer, a complete citrate substitution is unlikely. Additionally, citrate may form a secondary capping layer via hydrogen bonding,^34,35^ making total elimination of citrate interference nearly impossible. Nevertheless, with the aid of a deep learning model, differences between Pro and Hyp can be extracted, while residual citrate signals are treated as common features. To minimize interference from citrates, the data set obtained from AuNPs with monolayer capping and 48 h incubation was used for initial 1D-CNN model training.

## Deep Learning Analysis

**Figure 4:**
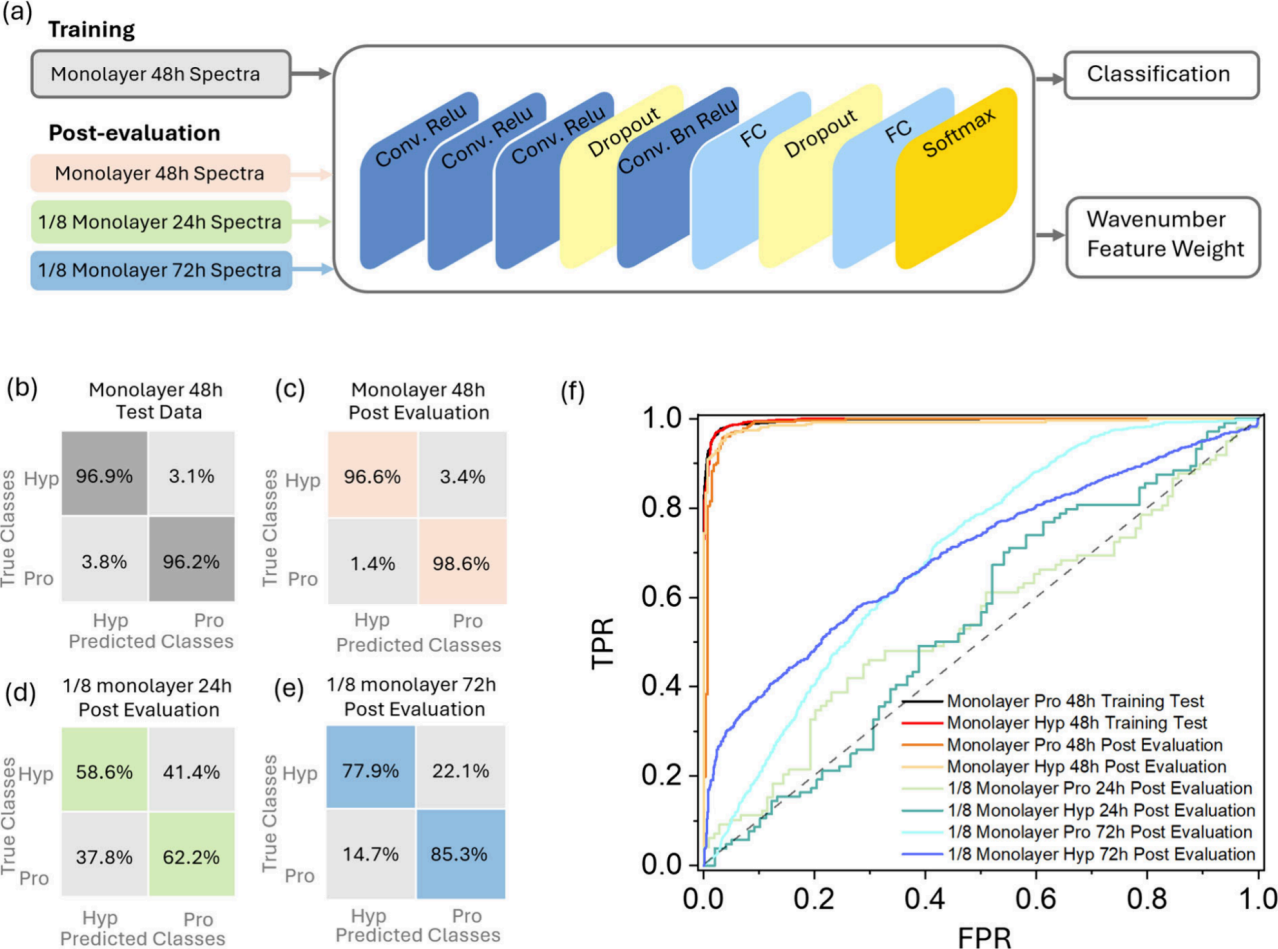
(a) Schematic representation of the CNN model architecture and the data classification framework. The confusion matrices illustrate the prediction accuracy for different data sets: (b) training data for monolayer Hyp and Pro after 48 h of incubation, (c) post evaluation of monolayer Hyp and Pro after 48 h of incubation, (d) post evaluation of 1/8 monolayer Hyp and Pro after 24 h of incubation, and (e) post evaluation of 1/8 monolayer Hyp and Pro after 72 h of incubation. (f) ROC curves for different data sets.

Deep learning-based models have been widely used in spectroscopy data analysis due to their self-adaptation characterization and high accuracy. A 1D-CNN model has been used for the prediction of Pro and Hyp.^36,37^ Here, we used MATLAB to run the codes of the 1D-CNN model for classification and post evaluation as shown in Figure 4(a). A total of 22,350 and 550 effective spectra with 1463 features were classified using 5-fold cross-validation for the CNN classification model and post evaluation. In the former, 80% of the spectra were used as the training set and 20% of the spectra were used as the test set, following these steps: (1) data set preparation, (2) training process, (3) evaluation metrics.

The model demonstrated accuracy higher than 96% in identifying single-molecule spectra of Pro and Hyp in the training set and post evaluation, as shown in Figure 4(b) and (c). It also showed tolerance to citrate interference by discriminating the data obtained under different incubation conditions. For the 72 h incubation and 1/8 monolayer coverage data set, the post evaluation accuracy is 77.9% and 85.3% for Hyp and Pro, respectively, in Figure 4(e). The lowering of the accuracy compared with the monolayer Pro or Hyp data set might be due to the interference of a larger amount of citrates remaining on AuNPs. However, the 24h 1/8 monolayer Pro and Hyp data set showed an accuracy of less than 70% in Figure 4(d), because the portion of citrate signals dominated the data set, which was also indicated by the peak occurrence frequency red curve in Figure 3(a) and histogram in Figure 3(b). Finally, the receiver operating characteristic (ROC) curves of the monolayer 48 h post evaluation of Pro and Hyp in Figure 4(f) show both high sensitivity and high specificity of the model in identifying new single-molecule spectra of Pro and Hyp.

## Interpretation of the CNN Output

**Figure 5:**
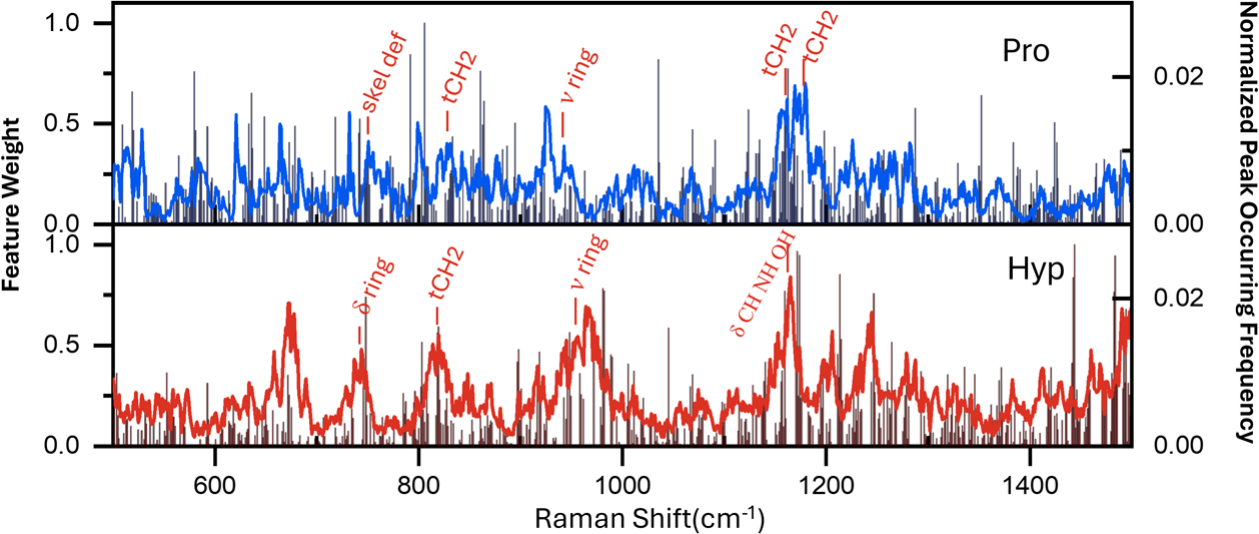
Feature weight extracted from the 1-CNN model. The blue and red curves show the normalized peak occurring frequency of a monolayer of Pro and Hyp with 48 h of incubation, respectively.

The 1D gradient-weighted feature visualization has become an emerging tool frequently used in recent works to extract spectral features from CNN models.^38,39^ However, many previous studies have applied this method to analyze multimolecule data with subtle spectral changes, rather than single-molecule data, which is inherently inhomogeneous.^38,39^ In this work, we extended its application to single-molecule spectral analysis and compared the extracted feature map of average spectra of the whole data set with the peak occurrence frequency to understand the performance of the 1D-CNN model. Figure 5 compares the positive feature weights extracted from the trained 1D-CNN model with the normalized peak occurrence frequency in the 600–1500 cm^–1^ region. The positive feature weights show narrow spikes per Raman shift (cm^–1^), indicating the high importance of the Raman bands for discrimination. We observed that high positive feature weight spikes (≥0.4) occur in the regions 750–800 cm^–1^, 800–825 cm^–1^, 900–1000 cm^–1^, and 1150–1200 cm^–1^, which correspond to band differences induced by structural changes. Table 1 lists the SERS shift and vibration modes for Pro and Hyp. In the 750–800 cm^–1^ region, the Hyp ring vibration band appears at 740 cm^–1^, while Pro shows a ring vibration-related band at 750 cm^–1^.^20,29^ In the region 800–825 cm^–1^, Hyp and Pro show a peak at 817 cm^–1^ and 824 cm^–1^, which corresponds to the ring fragment CH_2_ movement.^21^ The shift from 817 to 824 cm^–1^ is attributed to the structural change induced by the OH group on the ring. Similarly, at the region 900–1000 cm^–1^, Hyp and Pro show peaks at 943 cm^–1^ and 955 cm^–1^, respectively, corresponding to the ν_Ring_ mode.^21^ In the 1150–1200 cm^–1^ region, Hyp shows peaks at 1165 cm^–1^, respectively, corresponding to δ_CH NH OH_,^20^ while Pro shows peaks at 1159 cm^–1^ and 1174 cm^–1^ corresponding to t_CH2._^29^ Interestingly, the 1D-CNN model also gives high positive feature weight at positions with no peak occurrence, for example, the different valley at 1036 cm^–1^ for Pro and 1044 cm^–1^ and 1208 cm^–1^ for Hyp. Therefore, many highly weighted bands are aligned with the SERS bands with high occurring frequencies, indicating that the major contributions to the 1D-CNN performance originate from the difference in molecular structure between Hyp and Pro.

**Table 1:** SERS Bands Having a High Feature Weight in Figure 5 and the Corresponding Vibration Mode

Pro Band (cm^–1^)	Vibration Mode	Reference
750	skel deformation	(29)
824	t CH2	(21)
943	ν ring	(21)
1157, 1174	t CH2	(29)

Hyp Band (cm^–1^)	Vibration Mode	Reference
740	δ ring or ring torsion	(20)
817	t CH2	(21)
955	ν ring	(21)
1163	δ CH NH OH	(20)

In summary, we demonstrate, for the first time, the discrimination of proline and hydroxyproline at the single-molecule level using the particle-in-pore sensor assisted by the 1D-CNN model. The general characteristics of the single-molecule data set were obtained by calculating the peak occurrence frequency and plotting the frequency distribution. Using the peak occurrence frequency diagrams, we investigated the substitution of citrates by the analyte on AuNPs, revealing that sufficient incubation with the analyte solution reduced citrate bands in the spectra, indicating successful substitution. With the aid of a 1D-CNN model, we achieved more than 96% accuracy in discriminating proline from hydroxyproline. Even with 1/8 monolayer Pro and Hyp coverage, the discrimination accuracy was above 77%, demonstrating that the trained CNN model can tolerate some citrate interference. However, when excessive citrate remains on the AuNP surface, such as in the 1/8 monolayer, 24 h incubation data set, the 1D CNN model fails to correctly distinguish proline from hydroxyproline. The 1D gradient-weighted positive feature visualization was used to output normalized feature gradients, which were compared to peak occurrence frequencies. High positive feature weights were assigned to regions corresponding to vibration modes related to ring vibrations and OH bending. The successful discrimination of proline and hydroxyproline—one of the most challenging PTMs to detect at the single-molecule level—demonstrates the potential of our technology for various PTM discriminations. The single-molecule sensitivity of our method for detecting slight structural changes, including those with small Raman cross sections, also paves the way for site-specific PTM analysis in low-abundance peptides and proteins.

## Supporting Information Available

Materials, fabrication, and CNN model; distribution of nanopore size; schematic illustration of the microfluidic device; DLS size distribution of AuNP; calculation of amino acids and peptide concentration; citrate substitution on AuNP; Raman spectra of Pro and Hyp powder; AUC obtained from the CNN model (PDF)
