# Supplementary material for: Single-Molecule SERS Discrimination of Proline from Hydroxyproline Assisted by a Deep Learning Model
Source: Nano Lett. 2025 Apr 17;25(18):7499–506. doi: 10.1021/acs.nanolett.5c01177 (PMC12063160; doi:10.1021/acs.nanolett.5c01177)
Supplement: Supplementary file 1 — nl5c01177_si_001.pdf [file nl5c01177_si_001.pdf]

Supporting Information

Single-Molecule SERS Discrimination of Proline from Hydroxyproline Assisted by a Deep Learning Model

Yingqi Zhao,[1,3] Kuo Zhan,[1,3] Pei-Lin Xin,[1,3] Zuyan Chen,[4] Shuai Li,[4] Francesco De Angelis[5] and Jian-An Huang[1,2,3,*]

[1] Research Unit of Health Sciences and Technology, Faculty of Medicine, University of Oulu, Aapistie 5 A, 90220 Oulu, Finland.

[2] Research Unit of Disease Networks, Faculty of Biochemistry and Molecular Medicine, University of Oulu, Aapistie 5 A, 90220 Oulu, Finland.

[3] Biocenter Oulu, University of Oulu, Aapistie 5 A, 90220 Oulu, Finland.

4 The Biomimetics and Intelligent Systems (BISG) research unit, Faculty of Information Technology and Electronic Engineering, University of Oulu, Oulu, Finland

5 Istituto Italiano di Tecnologia, Via Morego 30, 16163, Genoa, Italy.

*Email: jianan.huang@oulu.fi

Supplementary Note 1. Materials, Fabrication and CNN model

Materials

Nonfunctionalized gold nanoparticles (AuNPs) with an average particle size of 50 nm from Sigma(753645-25ML, concentration of 3.5 × 10^{10} particles/mL). SYLGARD™ 184 Silicone Elastomer Kit was used for Polydimethylsiloxane (PDMS) microfluidic channel fabrication. Si wafers with 100nm SiN membranes coated on the surface were purchased from MicroChemicals GmbH. Trans-4-Hydroxy-L-proline(H54409) and L-Proline were purchased from Sigma.

Nanopore Device Fabrication

The gold nanoholes were fabricated on low-stress SiN membranes supported on silicon. The size and thickness of the SiN window were 1 × 1mm and 100nm, respectively. The size of silicon chips which support the SiN window is 1cm ×1 cm. After sputtering a 2 nm titanium and 100 nm gold layer on the front side and 20nm gold layer on the back side of the SiN membrane, focused ion beam(FIB) milling (FEI Helios DualBeam) from the back side of the membrane to create nanopores with average 200nm diameter. A scanning electron microscope(SEM) was used to characterise nanopore size and morphology from the front side. The nanopore distribution is shown in Figure S1, which was measured by SEM images from 3 different chips with, nine 9 pores on each chip. Then, the nanopore samples were embedded in a homemade microfluidic chamber made from PDMS. The schematic plot of the microfluidic device is shown in Figure S2.

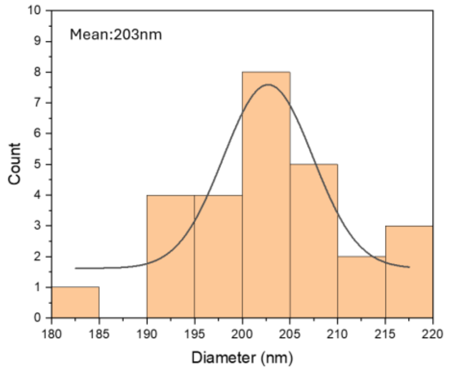

Figure S1. Size distribution of 27 nanopore

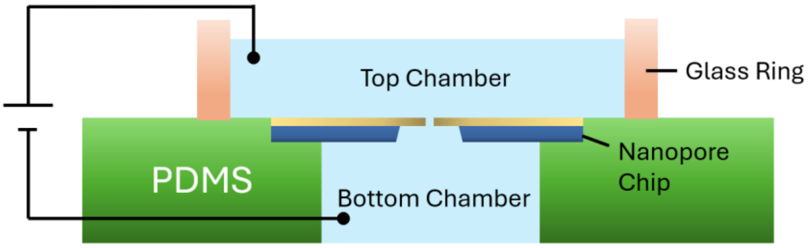

Figure S2. Schematic illustration of the microfluidic device.

Attachment of amino acid to AuNPs

All the amino acids used in the measurements were attached physically to gold nanoparticles, following the protocol in our previous work.[1,2] In the final solution for Raman measurements, the concentration of AuNPs and the salt concentration were 1.3×10^{10} particles per mL and 5% of pH 5.5 PBS buffer. Amino acid or peptide stock solutions were mixed with gold nanoparticles and PBS buffer. Before Raman measurement, the mixture was kept in a refrigerator at 4 °C for 48h to allow the adsorption of analytes on AuNP. The concentrations of amino acids and peptides in the final solution were calculated according to previous literature[3] To ensure the number of molecules adsorbed on each AuNP forms a monolayer, in the particle-in-pore system, only one molecule occupies the hot spot and generates a single-molecule SERS signal. The details of the concentration calculation are discussed in the Supplementary Note 2. According to dynamic light scattering (DLS), the average particle size of the as-purchased AuNP in 5% PBS at pH 5.5 was 59 nm. After Pro or Hyp incubation for 48 hours, the particle size remained at 59 nm. The distribution of particle size of AuNP before and after incubation in Pro and Hyp solutions is shown in Figure S3 below.

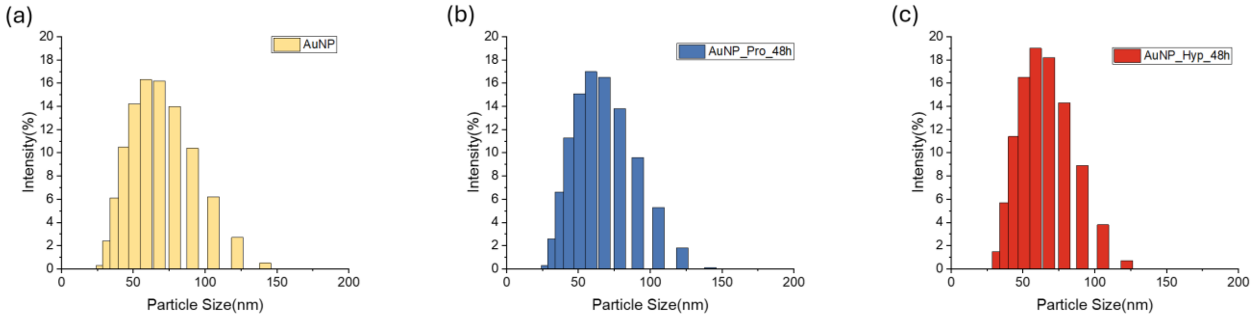

Figure S3. The DLS size distribution of (a)pure AuNP, (b)AuNP incubated for 48h in Pro solution for monolayer capping, and (c)AuNP incubated for 48h in Hyp solution for monolayer capping.

Raman measurement

Raman measurement was performed using a Thermo Scientific DXR2xi Raman Imaging Microscope under a 785nm laser with a 60x water immersion objective. The spectra accumulation time was 0.1s, and the laser power was 10 mW. The time series Raman spectra were collected by Andor Solis. Before measurement, the nanopore devices were treated with O_2 plasma to generate a hydrophilic surface and pore side walls. Then AuNP was loaded with the analyte, and 5% PBS with pH 5.5 was filled in the bottom and top chambers of the nanopore device. Proline and hydroxyproline were tested in different devices. A new sensor device was used for each AuNP incubation time. During the measurement, a 0-0.6V cross-SiN membrane voltage was applied using platinum electrodes to facilitate the gold nanoparticle trapping in the nanopore. Signals were collected from more than five different nanopores on the same device.

Raman Data processing

Raman data analysis was performed using MATLAB R2022a software. All the raw data were pre-processed by performing cosmic ray removal, normalization, and baseline reduction. Peak findings were then carried out to locate the peak position using the “find peak” function in MATLAB. A minimum peak intensity threshold of 0.07 was set to select the peak from the noise. The spectra with found peaks were regarded as effective and recorded for further analysis. The number of raw spectra and effective spectra are listed in the form S2 in the supporting information. The normalized peak

occurring frequency was calculated by counting the peak occurring at each Raman shift and dividing by the number of effective spectra.

Table S1. Number of Effective Spectra after Pre-processing

Molecule on AuNP	Analyte coverage on AuNP	Incubation time （hour）	Number of Effective spectra
Citrate	Monolayer	0	8807
Pro	Monolayer	48	9769
Hyp	Monolayer	48	11002
Pro	1/8 Monolayer	24	11001
Hyp	1/8 Monolayer	24	9768
Pro	1/8 Monolayer	72	15284
Hyp	1/8 Monolayer	72	10345

CNN Model

The detailed codes are seen in Github link:

https://github.com/KuoZHAN/Discrimination-of-Proline-and-Hydroxylated-Proline

1D gradient-weighted feature visualization was adopted to extract outputs normalized feature gradients, providing insights into the role of specific features in model predictions. The approach utilizes feature gradients to quantify the contribution of individual features to a trained CNN model, facilitating interpretability and model diagnostics. Following these steps: 1. Pre-Trained Network Modification The trained CNN model was loaded, and its architecture was modified to allow gradient computation. 2. Data Preparation Features for the first sample (1:1463) were extracted and normalized using pre-computed normalization parameters 3. Gradient computation. Gradients of the loss concerning input features were calculated for each sample. The loss function was defined as the categorical cross-entropy between predicted and true labels. Gradients were computed using a custom function. The detailed codes are seen in the supporting information.

Supplementary Note 2. Calculation of amino acids (AA) and peptide concentration

The concentration of AA required to achieve monolayers on the gold nanoparticle (AuNP) surfaces was determined by empirical values of maximum solvent accessibilities[3] of residues in proteins found in the literature[2] . The surface area of a single ϕ50 nm AuNP is calculated as 7850 nm^2. The amount of AAs molecules required to form a monolayer on a single AuNP was calculated. Then we determined the final molecule concentration, taking account of the AuNP concentration of 1.3×10^{10} mL^{-1}. The Maximum accessible surface area of Pro is 154$Å^2$,[3] the number of molecules per AuNP to form a monolayer is 5097, resulting in final Pro concentration of incubation solution 105nM. The same Hyp concentration was used.

Supplementary Note 3. The citrate substitution on AuNP

The binding Gibbs free energies between the molecule and metal were used to characterize the affinity of molecule adsorption on metal nanoparticles.[4] According to theoretical calculation, the Gibbs free energies of amino acids bond gold are lower than -60 kcal/mol.[4] At pH 5.5, most citrate shows the zwitterion form, which has an average of -20 kcal/mol on the gold surface according to simulation.[5] Therefore, the amino acid adsorbs to AuNP with priority and substitutes the citrate in the incubation solution with pH5.5.

In the previous literature,[6] high concentrations of high-affinity ions, for example, millimolar levels of I^-, can sufficiently substitute the citrate on AuNP. However in our system, to ensure a monolayer of analyte capping on the AuNP, the analyte concentration was only 105nM, therefore the substitution may be insufficient. Also according to previous literature,[7, 8] citrate may re-adsorb on the surface of AuNP. Therefore, the possibility of citrate existence is difficult to fully preclude. With the assistance of a deep learning model, the citrate can be treated as a common feature while discriminate the analyte difference.

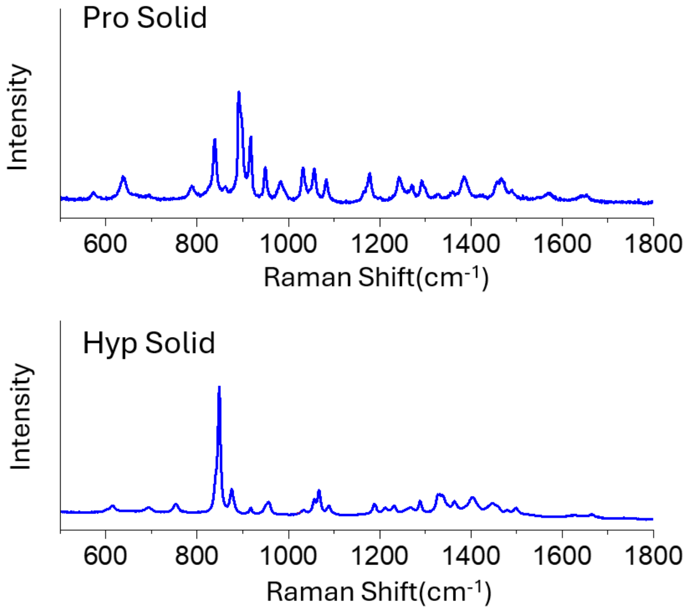

Figure S4. Raman spectra of Pro and Hyp powder.

Table S2. The AUC Obtained from the CNN Model

Data Set	AUC
Training data for monolayer Pro 48h	0.98894
Training data for monolayer Hyp 48h	0.98913
Post evaluation of monolayer Pro 48h	0.98614
Post evaluation of monolayer Hyp 48h	0.98612
Post evaluation of 1/8 monolayer Pro 24h	0.5631
Post evaluation of 1/8 monolayer Hyp 24h	0.5627
Post evaluation of 1/8 monolayer Pro 72h	0.69731
Post evaluation of 1/8 monolayer Hyp 72h	0.69698

Reference

(1) Huang, J.-A.; Mousavi, M. Z.; Zhao, Y.; Hubarevich, A.; Omeis, F.; Giovannini, G.; Schütte, M.; Garoli, D.; De Angelis, F. SERS discrimination of single DNA bases in single oligonucleotides by electro-plasmonic trapping. *Nature Communications* **2019**, *10* (1), 5321. DOI: 10.1038/s41467-019-13242-x.
(2) Huang, J.-A.; Mousavi, M. Z.; Giovannini, G.; Zhao, Y.; Hubarevich, A.; Soler, M. A.; Rocchia, W.; Garoli, D.; De Angelis, F. Multiplexed Discrimination of Single Amino Acid Residues in Polypeptides in a Single SERS Hot Spot. *Angewandte Chemie International Edition* **2020**, *59* (28), 11423-11431. DOI: https://doi.org/10.1002/anie.202000489.
(3) Tien, M.; Meyer, A.; Sydykova, D.; Spielman, S.; Wilke, C. Maximum Allowed Solvent Accessibilites of Residues in Proteins. *Plos One* **2013**, *8* (11), e80635, Article. DOI: 10.1371/journal.pone.0080635.
(4) Buglak, A.; Kononov, A. Comparative study of gold and silver interactions with amino acids and nucleobases. *Rsc Advances* **2020**, *10* (56), 34149-34160, Article. DOI: 10.1039/d0ra06486f.
(5) Franco-Ulloa, S.; Tatulli, G.; Bore, S.; Moglianetti, M.; Pompa, P.; Cascella, M.; De Vivo, M. Dispersion state phase diagram of citrate-coated metallic nanoparticles in saline solutions. *Nature Communications* **2020**, *11* (1), 5422, Article. DOI: 10.1038/s41467-020-19164-3.
(6) Xu, L.; Zong, C.; Zheng, X.; Hu, P.; Feng, J.; Ren, B. Label-Free Detection of Native Proteins by Surface-Enhanced Raman Spectroscopy Using Iodide-Modified Nanoparticles. *Analytical Chemistry* **2014**, *86* (4), 2238-2245, Article. DOI: 10.1021/ac403974n.
(7) Park, J.; Shumaker-Parry, J. Structural Study of Citrate Layers on Gold Nanoparticles: Role of Intermolecular Interactions in Stabilizing Nanoparticles. *Journal of the American Chemical Society* **2014**, *136* (5), 1907-1921, Article. DOI: 10.1021/ja4097384.
(8) Majzik, A.; Fülöp, L.; Csapó, E.; Bogár, F.; Martinek, T.; Penke, B.; Bíró, G.; Dékány, I. Functionalization of gold nanoparticles with amino acid, β-amyloid peptides and fragment. *Colloids and Surfaces B-Biointerfaces* **2010**, *81* (1), 235-241, Article. DOI: 10.1016/j.colsurfb.2010.07.011.
